# Radiation therapy before radical cystectomy combined with immunotherapy in locally advanced bladder cancer – study protocol of a prospective, single arm, multicenter phase II trial (RACE IT)

**DOI:** 10.1186/s12885-019-6503-6

**Published:** 2020-01-03

**Authors:** Sebastian C. Schmid, Florestan J. Koll, Claus Rödel, Philipp Maisch, Andreas Sauter, Franziska Beckert, Anna Seitz, Hubert Kübler, Michael Flentje, Felix Chun, Stephanie E. Combs, Kilian Schiller, Jürgen E. Gschwend, Margitta Retz

**Affiliations:** 1 Department of Urology, School of Medicine, Rechts der Isar Medical Center, Technical University of Munich, Ismaninger Straße 22, 81675 Munich, Germany; 2 Department of Radiation Oncology, University of Frankfurt, Frankfurt, Germany; 3 Department of Diagnostic and Interventional Radiology, School of Medicine, Rechts der Isar Medical Center, Technical University of Munich, Munich, Germany; 4 Department of Urology, University of Würzburg, Würzburg, Germany; 5 Department of Radiation Oncology, University of Würzburg, Würzburg, Germany; 6 Department of Urology, University of Frankfurt, Frankfurt, Germany; 7 Department of Radiation Oncology, School of Medicine, Rechts der Isar Medical Center, Technical University of Munich, Munich, Germany; 8 Helmholtz Zentrum München (HMGU), Institute of Radiation Medicine (IRM), Deutsches Konsortium für Translationale Krebsforschung (NeoDKTK) Partner Site Munich, Oberschleißheim, Germany

**Keywords:** Bladder cancer, Urothelial cancer, Transitional cell carcinoma, Locally advanced, Immunotherapy, Radiotherapy, Radical cystectomy, Nivolumab, Checkpoint inhibitor, PD-1 inhibitor

## Abstract

**Background:**

Patients with locally advanced bladder cancer (cT3/4 cN0/N+ cM0) have a poor prognosis despite radical surgical therapy and perioperative chemotherapy. Preliminary data suggest that the combination of radiation and immunotherapy does not lead to excess toxicity and may have synergistic (abscopal) anti-tumor effects. We hypothesize that the combined preoperative application of the PD-1 checkpoint-inhibitor Nivolumab with concomitant radiation therapy of the bladder and pelvic region followed by radical cystectomy with standardized lymphadenectomy is safe and feasible and might improve outcome for patients with locally advanced bladder cancer.

**Methods:**

**Study design: “**RACE IT” (AUO AB 65/18) is an investigator initiated, prospective, multicenter, open, single arm phase II trial sponsored by Technical University Munich. Study drug and funding are provided by the company Bristol-Myers Squibb.

**Study treatment:** Patients will receive Nivolumab 240 mg i.v. every 2 weeks for 4 cycles preoperatively with concomitant radiation therapy of bladder and pelvic region (max. 50.4 Gy). Radical cystectomy with standardized bilateral pelvic lymphadenectomy will be performed between week 11–15.

**Primary endpoint:** Rate of patients with completed treatment consisting of radio-immunotherapy and radical cystectomy at the end of week 15.

**Secondary endpoints:** Acute and late toxicity, therapy response and survival (1 year follow up).

**Main inclusion criteria:** Patients with histologically confirmed, locally advanced bladder cancer (cT3/4, cN0/N+), who are ineligible for neoadjuvant, cisplatin-based chemotherapy or who refuse neoadjuvant chemotherapy.

**Main exclusion criteria:** Patients with metastatic disease (lymph node metastasis outside pelvis or distant metastasis) or previous chemo-, immune- or radiation therapy.

**Planned sample size:** 33 patients, interim analysis after 11 patients.

**Discussion:**

This trial aims to evaluate the safety and feasibility of the combined approach of preoperative PD-1 checkpoint-inhibitor therapy with concomitant radiation of bladder and pelvic region followed by radical cystectomy. The secondary objectives of therapy response and survival are thought to provide preliminary data for further clinical evaluation after successful completion of this trial. Recruitment has started in February 2019.

**Trial registration:**

Protocol Code RACE IT: AB 65/18; EudraCT: 2018–001823-38; Clinicaltrials.gov: NCT03529890; Date of registration: 27 June 2018.

## Background

Bladder cancer is the 9th most common cancer worldwide with about 430,000 new cases each year. About 25% of patients present with muscle-invasive disease at the time of diagnosis [1]. The current standard of care for muscle-invasive bladder cancer (MIBC) is radical cystectomy with pelvic lymphadenectomy. According to German and European guidelines, neoadjuvant chemotherapy is recommended for patients with MIBC, who are fit to receive cisplatin-based chemotherapy [2, 3]. Unfortunately, around 50% of patients are ineligible to receive neoadjuvant chemotherapy mainly because of impaired renal function [4].

Patients with locally advanced bladder cancer (cT3/4 cN0/N+ cM0) have a poor prognosis despite radical surgical therapy and systemic treatment. If tumor invades perivesical tissue (pT3), 5-year overall survival (OS) is about 43% and drops as low as 28% in case of infiltration of surrounding tissue (pT4). If tumor has spread to local lymph nodes, only every 5th patient will survive 5 years after surgery [5]. The addition of perioperative chemotherapy only adds a small but significant absolute survival benefit to surgery alone in patients with MIBC [6, 7].

Immune checkpoint-inhibitors have shown impressive results in clinical trials in advanced bladder cancer, leading to FDA and EMA approval as first and second line therapy in metastatic urothelial cancer. Targeting the immune checkpoints “programmed death ligand-1” (PD-L1), “programmed cell death protein-1” (PD-1) and “cytotoxic T-lymphocyte associated protein 4” (CTLA-4) with antibodies leads to T-cell activation and anti-tumor immune response [8]. In Europe the PD-1/PD-L1 inhibitors Nivolumab, Pembrolizumab and Atezolizumab are approved for metastatic bladder cancer [9, 10]. The PD-1 inhibitor Nivolumab was analyzed in the single arm, phase II CheckMate 275 trial, which included 270 evaluable patients with progressive metastatic urothelial cancer after cisplatin-based chemotherapy. Confirmed objective response was achieved in about 20% of patients. Grade 3–4 treatment-related adverse events occurred in 48 (18%) of 270 patients-most commonly grade 3 fatigue and diarrhea. Five deaths were attributed to treatment (pneumonitis, acute respiratory failure, multifactorial acute respiratory failure, septic shock, and cardiovascular failure) [11]. Two current trials are evaluating immune-checkpoint blockade in a neoadjuvant setting (NCT02736266 and NCT02662309) with promising early results [12, 13].

In regard to radiation therapy, neoadjuvant radio (chemo) therapy (RCHT) has shown its efficacy in other tumor entities such as esophageal or colorectal carcinoma [14–16]. In bladder cancer, the sequence of preoperative radio (chemo) therapy (RCHT) followed by radical cystectomy is a common therapy pathway in the setting of trimodal therapy (TMT), which is an accepted alternative treatment for MIBC according to the German S3-guideline [3]. There is promising retrospective data for neoadjuvant RCHT in locally advanced bladder cancer [17]. Since recent preclinical and early clinical trials propose a synergistic effect of radiation and immunotherapy, this combination seems to be an interesting alternative to RCHT [18, 19]. Radiotherapy can lead to immunogenic cell death, which leads to the release and presentation of tumor antigens, which in turn can lead to priming and activation of T-cells. Furthermore, radiotherapy induces antigen presentation and cytokine release of the tumor, which further leads to T-cell recruitment. On the other hand, radiation can induce increased PD-L1 expression in the tumor, hindering the efficiency of attracted T-cells [20]. Currently various clinical phase II and phase III trials explore the combination of radiation therapy and PD-1 inhibition in different tumor entities [18]. Concomitant treatment with PD-L1/PD-1 inhibitors led not only to partial or complete remissions, but also to abscopal (outside the radiation field) effects [20]. Therefore, combined application of radiotherapy with Nivolumab before radical cystectomy might lead to improved cure rates and local control in this otherwise poor prognostic subgroup with locally advanced bladder cancer. Notably, this treatment can be given irrespective of kidney function, which is impaired in 30–50% of these patients [21].

### Aims

The primary objective of RACE IT is to evaluate safety and feasibility of the combined application of preoperative radiation therapy with the PD-1 checkpoint-inhibitor Nivolumab followed by radical cystectomy in patients with locally advanced bladder cancer. We secondarily hypothesize that the combined application of preoperative radiation therapy with the PD-1 checkpoint-inhibitor Nivolumab before radical cystectomy leads to improved disease-free survival (DFS) and overall survival (OS) compared to historical controls.

## Methods and study-design

### Study design

RACE IT is a prospective, multicenter, open, single arm phase II study.

### Inclusion and exclusion criteria

Patients with histologically confirmed, locally advanced bladder cancer (cT3/4, cN0/N+), who are ineligible for neoadjuvant, cisplatin-based chemotherapy or who refuse neoadjuvant chemotherapy can be included in this study. Main exclusion criteria are metastatic disease (lymph node metastasis outside pelvis or distant metastasis) or previous chemo-, immune- or radiation therapy. All inclusion and exclusion criteria are shown in Table 1.

**Table 1:** Inclusion and exclusion criteria

Criteria	Details
Inclusion	- Histologically confirmed, locally advanced bladder cancer (cT3/4 cN0/N+ cM0) with ≥10% urothelial differentiation - Ineligibility for neoadjuvant cisplatin-based chemotherapy due to any of the following criteria: • Creatinine Clearance (using the Cockcroft-Gault formula) < 60 mL/min • Hearing loss ≥ grade 2 (CTCAE version 4) • Peripheral neuropathy ≥ grade 2 (CTCAE version 4) • ECOG performance score 2 - Subjects that are eligible for cisplatin may be candidates if they refuse available neoadjuvant cisplatin-based chemotherapy, despite being informed by the investigator about the treatment options. The subject’s refusal must be thoroughly documented. - ECOG 0–2 - Life expectancy > 6 months - Adequate function of bone marrow, liver and coagulation as determined by blood tests - Able to give informed consent - Body weight 35 kg - 160 kg
Exclusion	- Metastatic disease defined as distant metastasis or suspicious lymph nodes outside the pelvis (above aortic bifurcation) using RECIST 1.1 criteria - Prior chemotherapy before treatment (not including intravesical chemotherapy) - Prior radiation therapy of the pelvis - Active, known or suspected autoimmune disease (not including: vitiligo, allergic rhinitis/asthma, type 1 diabetes mellitus, residual hypothyroidism due to an autoimmune condition only requiring hormone replacement, psoriasis not requiring systemic treatment, or conditions not expected to recur in the absence of an external trigger) - Immunosuppressive treatment with corticosteroids or other drugs within 14 days of study drug administration (not including: inhaled or topical steroids and adrenal replacement doses are permitted in the absence of active autoimmune disease) - Experimental therapy or clinical trial at time of inclusion or the previous 4 weeks before inclusion - Previous treatment with an anti-PD-1, anti-PD-L1, anti-PD-L2, anti-CTLA-4 antibody, or any other antibody or drug specifically targeting T-cell costimulation or immune checkpoint pathways (not including BCG therapy) - Any uncontrolled or severe cardiovascular or pulmonary disease determined by the investigator, including i) NYHA functional classification III or IV, congestive heart failure, unstable or poorly controlled angina, uncontrolled hypertension, serious arrhythmia or myocardial infarction in the past 12 months before inclusion; ii) Subjects with interstitial lung disease that is symptomatic or may interfere with the detection or management of suspected drug-related pulmonary toxicity. - End-stage kidney disease defined as GFR < 15 mL/min or need for dialysis - Thromboembolic events like pulmonary embolism or stroke in previous 3 months before inclusion - Other active tumor disease (not including basal cell carcinoma of the skin and carcinoma in situ of the cervix). Tumor is regarded non-active after curative therapy and 5 years of follow up without pathological findings. - Medium to extended surgery or trauma in the previous 4 weeks before inclusion (not including transurethral bladder resection, nephrostomy or ureteral stent or biopsy) - Uncontrolled and serious somatic or mental illness - Pregnant or lactating women - Age < 18 years - Prior organ transplantation - Positive test result for hepatitis B or C indicating acute or chronic infection - Positive HIV test or acquired immunodeficiency syndrome (AIDS) - Gastrointestinal disorders, particularly those with high risk of perforation or fistula formation including i) active peptic ulcer disease or active inflammatory bowel disease (incl. Ulcerative colitis and Crohn’s disease), diverticulitis, cholecystitis, symptomatic cholangitis or appendicitis) during screening and/or ii) history of abdominal fistula or bowel perforation within 6 months prior to first dose of study treatment.

### Endpoints

#### Primary endpoints

Rate of patients with completed treatment consisting of radio-immunotherapy and radical cystectomy at the end of week 15. Completed treatment is defined by administration of at least two complete cycles of Nivolumab with 240 mg i.v. and administration of at least 23 of planned 28 radiation fractions (≥ 41.4 Gy).

#### Secondary endpoints

- Acute toxicity of preoperative radio-immunotherapy followed by radical cystectomy until 3 months after end of therapy according to CTCAE v4. Typical, predefined side effects of surgery will be excluded from analysis.
- Rate of immune related toxicities: Immune mediated pneumonitis, colitis, hepatitis, hypophysitis, adrenal insufficiency, hypo−/hyperthyroidism, diabetes (type 1), nephritis, immune mediated skin reactions
- Late toxicity during 1 year follow up according to CTCAE v4
- Disease free survival (DFS) defined by local recurrence or distant metastasis or death in R0 resected patients during 1 year follow up starting at the date of cystectomy
- Time to death by any cause during 1 year follow up (overall survival (OS)) starting at the date of cystectomy
- Radiological overall response rate after radio-immunotherapy before radical cystectomy (complete response, partial response, stable disease, progressive disease)
- ypT0 rate after radical cystectomy
- Surgical margin status after cystectomy (R0/R1/R2)

### Treatment and follow up

Treatment starts after successful completion of the screening phase and confirmation of eligibility. Nivolumab 240 mg flat dose will be given intravenously on day 1. Nivolumab will be given every 2 weeks for a total of 4 cycles. The last infusion will be in week 7. Standard radiation therapy will start on day 8 (week 2). The radiation therapy will be given in 28 fractions over approximately 5 and a half weeks. The total dose is 50.4 Gy in fractions of 1.8 Gy per day with 45 Gy of the pelvic region and a consecutive boost of bladder/tumor with 5.4 Gy. Imaging will be performed before start of treatment as baseline and repeated after finishing of study treatment before radical cystectomy to rule out progression or systemic disease. In case of systemic disease, no cystectomy will be performed in curative intention. The surgery consisting of open radical cystectomy with urinary diversion (ileum conduit or neobladder) and standardized pelvic lymphadenectomy will be performed within week 11–15. The standardized lymphadenectomy is exactly defined in a SOP and includes the external and internal iliac region, obturator fossa as well as commune iliac region.

**Fig. 1:**
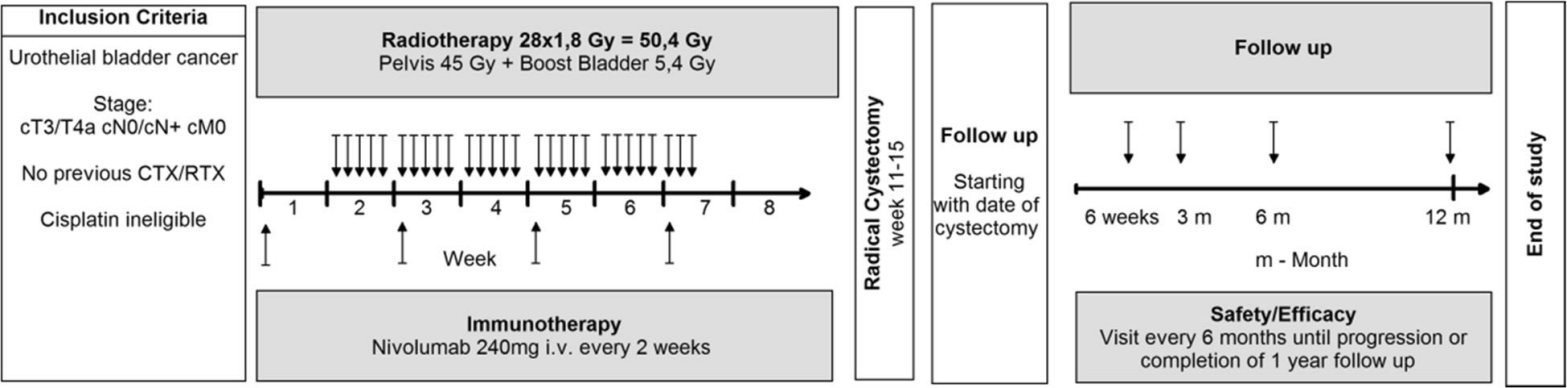
Schematic outline of the treatment plan. Patients with locally advanced bladder cancer, included in RACE IT study, will receive Nivolumab 240 mg i.v. every 2 weeks for 4 cycles preoperatively with concomitant radiation therapy of bladder and pelvic region (max. 50.4 Gy) Radical cystectomy with standardized bilateral pelvic lymphadenectomy will be performed between week 11–15. Follow up for secondary endpoints will be starting at the date of cystectomy for 1 year every 6 months

Follow up for secondary endpoints will be starting at the date of cystectomy for 1 year (52 weeks). A schematic outline of the treatment plan is shown in Fig. 1.

Basis of the follow up is the German bladder cancer guideline. Post-surgery visits at 6 and 12 weeks and 6 and 12 months after date of cystectomy include survival status, urine dipstick test, laboratory tests as well assessment of cystectomy histology and details of surgery (week 6 postop), assessment of subsequent therapy after surgery, symptoms, Adverse Event (AE), concomitant medication, ECOG score, ultrasound of both kidneys and a symptom oriented physical examination. A CT-scan of thorax, abdomen and pelvis with i.v. contrast will be performed at month 6 and 12. MRT of abdomen/pelvis with native CT thorax can be used in case of impaired kidney function or contrast medium allergies. The quality of life will be assessed at the screening-visit, pre-surgery and 3, 6 and 12 months after surgery with questionnaires (EORTC QoL-C30, ICIQ-SF, IIEF-5/6 (male) und FSFI-19 (female), FACT-Bl). A detailed flowchart for minimum assessments during treatment and follow up phase is shown in Table 2.

**Table 2:** Flowchart for minimum assessments of treatment and follow up phase

	Study Phase	Screening	Treatment visit	Treatment visit	Treatment visit	Treatment visit	Treatment visit		Postop visit	Postop visit	Follow up visit	Follow up visit
	Therapy		Nivolumab cycle 1	Nivolumab cycle 2	Nivolumab cycle 3	Nivolumab cycle 4	End RIT/PreOP		Postop 6w	Postop 12w	Follow up 6 m postop	Follow up 12 m postop
	Timepoint	-d28 – d0	d1 (w1d1)	d15 (w3d1)	d29 (w5d1)	d43 (w7d1)	d64 (w10d1)		w17–21	w23–27	w37–41	w63–67
Procedures	Visit No	1	2	3	4	5	6		7	8	9	10
Informed Consent		X						Radical Cystectomy week 11–15				
Inclusion/Exclusion Criteria		X										
Medical History		X										
Prior cancer therapy		X										
ECOG/Performance Status		X	X	X	X	X	X		X	X	X	X
Physical Examination		X	X	X	X	X	X		X	X	X	X
Vital Signs		X	X	X	X	X	X		X	X		
Height/Weight		X	X	X	X	X	X		X	X		
Symptom/Adverse Event Assessment		X	X	X	X	X	X		X	X	X	X
Bladder/surgery specific AEs		X	X	X	X	X	X		X	X	X	X
Concomitant Medication		X	X	X	X	X	X		X	X		
Laboratory tests		X	X	X	X	X	X		X	X	X	X
HIV/Hepatitis test		X							X			
Pregnancy test (Serum)		X					X					
Urine analysis		X					X		X	X	X	X
Kidney Ultrasound		X							X	X	X	X
Imaging (CT/MRT)		X					X				X	X
Histology assessment		X							X			
QoL-Questionnaires		X					X			X	X	X
Exploratory biomarker blood draw		X					X			X		
Survival status									X	X	X	X
Review of subsequent therapy									X	X	X	X

### Adverse events

Adverse Events (AEs) will be collected during the complete study period (treatment and follow up). All severe adverse events (SAEs) will be collected starting with the screening period until visit 8 (12 weeks after surgery). AEs of interest are immune-mediated, which are specific events that include pneumonitis, diarrhea/colitis, hepatitis, nephritis/renal dysfunction, rash, and endocrine events (adrenal insufficiency, hypothyroidism/thyroiditis, hyperthyroidism, diabetes mellitus, and hypophysitis) for which subjects received immunosuppressive medication for treatment of the event, with the exception of endocrine events, which are included regardless of treatment since these events are often managed without immunosuppression.

In addition to the assessment of all AEs by CTCAE, surgery- and bladder related adverse events are explicitly asked for and collected in detail, including peri-operative and postoperative complications (such as revision surgery, transfusion, anastomotic stricture or insufficiency, ileus, sepsis, cardiovascular events and so on). The following typical, predefined side effects of surgery will be excluded from analysis: short term paralytic ileus post-surgery without need for intervention, short term reactive diarrhea post-surgery, short term and asymptomatic hydronephrosis without creatinine elevation post-surgery, bacterial colonization of indwelling catheters post-surgery.

### Statistical calculations for trial sample size

The estimations in regard to the primary endpoint are derived from data from neoadjuvant cisplatin-based chemotherapy followed by radical cystectomy as current standard of treatment. In this regimen, the rate of patients with completed treatment (neoadjuvant therapy and radical cystectomy) is between 90 and 95% [21]. Trials of neoadjuvant radiotherapy in urinary bladder cancer showed no additional postoperative toxicity [2, 3]. Comparing the toxicity of Nivolumab to cisplatin, we assume that a completion rate of 90–95% is a reasonable estimation for the neoadjuvant radio-immunotherapy.

Assuming a rate ≥ 92.5% of patients with completed treatment at the end of week 15, we would tolerate an additional 22.5% of treatment delay (≥ 70% patients with completed treatment at the end of week 15). Thirty patients will be required to reject the null-hypothesis of a rate < 70% patients with completed treatment at the end of week 15 with 82% power and a 2-sided significance level of 5%. Additional 3 patients will be enrolled to account for possible drop-outs. This leads to a panned sample size of 33 patients. Recruitment will be conducted during a period of 2 years.

### Interim analysis

To ensure patient safety, a planned interim analysis will be performed after 11 patients, with review of an independent data safety monitoring board.

### Data analysis

All continuous endpoints will be summarized using descriptive statistics. All categorical endpoints will be summarized using absolute frequencies and percentages.

For the primary endpoint, an exact test for single proportions will be performed to reject the null hypothesis of ≥22.5% of treatment-related delay in surgery at week 15. Proportions will be displayed together with their 90% confidence intervals. If the resulting *p*-value is less than 5%, the study is considered successful. The primary analysis is based on the Full Analysis Set (FAS). Kaplan-Meier curves will be used to describe event-free rates over time (DFS, CSS and OS). Median event-free times will be reported with 95% CI, if the number of events allows the estimation of the median.

### Ethics, informed consent and safety

This study is conducted in accordance with Good Clinical Practice (GCP), as defined by the International Conference on Harmonization (ICH) and in accordance with the ethical principles underlying European Union Directive 2001/20/EC. The study is conducted in compliance with the study-protocol. For the present study an EudraCT-Number (2018–001823-38) has been obtained. The final study protocol has been approved by the ethics committee of the Technical University of Munich, Germany (Protocol Number AB 65/18) as well as by the responsible German government authority Paul-Ehrlich-Institute, Langen, Germany.

Investigators will ensure that patients are clearly and fully informed about the purpose, potential risks, and other critical issues regarding clinical studies in which they volunteer to participate. The informed consent form will adhere to the ethical principles that have their origin in the Declaration of Helsinki. All collected data from patients with signed informed consent will be entered into the electronic Case Report Form by the investigator. Sponsor representatives will review data centrally to identify potential issues to determine a schedule of on-site visits for targeted review of study records. In addition, the study may be evaluated by sponsors internal auditors and government inspectors who must be allowed access to Case Report Form, source documents, other study files, and study facilities.

Data collection and management will be performed according to general data protection regulation of the European Union.

### Trial organization

RACE IT is an investigator-initiated trial. The sponsor is Technical University Munich, faculty for medicine, which is a German government funded university. The trial is funded by Bristol-Myers Squibb Company. The study drug Nivolumab is provided by Bristol-Myers Squibb Company. Other study sites will be University Hospital, Johann Wolfgang Goethe-Universität, Frankfurt, Germany and the University Hospital Würzburg, Germany.

## Discussion

RACE IT study aims to evaluate immunotherapy with the PD-1 checkpoint-inhibitor Nivolumab combined with radiation therapy followed by radical cystectomy for patients with locally advanced bladder cancer.

Survival rates of patients with locally advanced bladder cancer treated with radical cystectomy are poor. The addition of neoadjuvant chemotherapy could increase 5-year survival only about 5–8% [2, 6] indicating the need for novel additional therapies. Retrospective analyses have shown promising results for the use of RCHT in the neoadjuvant setting, with 5-year disease specific survival (DSS) of 62% compared to 27% in their historical “cystectomy only” cohort [17]. Around 50% of patients are ineligible to receive neoadjuvant chemotherapy mainly because of impaired renal function [4], thus ongoing trials evaluate the benefit of immune-checkpoint inhibitors in a neoadjuvant setting since they can be given regardless of kidney function. An Open-Label, Single-Arm, Phase II Study (PURE-01) analyzed the activity of pembrolizumab as neoadjuvant immunotherapy before radical cystectomy for MIBC and showed downstaging to pT < 2 in 54% of patients and 42% pT0-patients in RC after 3 cycles of pembrolizumab. Notably, all 55 patients enrolled in the study underwent radical cystectomy [22]. Three patients (6%) had grade 3 AEs (diarrhea, hyperkaliemia, ALT/AST-increase) that caused pembrolizumab discontinuation for one patient. Response rates were significantly dependent on the PD-L1 status (highly enriched in patients with PD-L1 CPS ≥ 10% vs. no appreciable antitumor effects patients with CPS < 10%) and significant association between tumor mutation burden (TMB) and pT0 was observed. Interim results of the ABACUS-trial showed that neoadjuvant atezolizumab is safe and associated with a significantly improved pathological CR (29%). Treatment related grade 3/4 toxicity occurred in 12% of patients and 7 out of 69 patients (10%) did not have cystectomy [13].

These studies show promising results with high rates of downstaging, but the responses were significantly dependent on the PD-L1 status [22]. Response rates might even be increased with concomitant radiation therapy, which leads to immunogenic cell death, release of T-cell attracting cytokines and upregulation of surface molecules. There is preliminary evidence to believe that the combination of radiation therapy with immunomodulating drugs has a promising potential for synergistic and off-target effects, without severe toxicities and might improve downstaging and operability of the tumor [18, 19]. We believe that response and survival rates might be improved due to the promising results of immunomodulating agents in MIBC and metastatic bladder cancer as well as the possible synergistic effects of radiation therapy and PD-1 inhibition.

One critical point of neoadjuvant therapy in general is the delay or even truncation of definitive therapy. This study holds a risk of delay of surgery and consecutive progression-risk for patients. In the PURE-01 trial the neoadjuvant administration of immunotherapy did not delay planned surgery [22]. Available data and clinical experience suggest, that the combination of radiation and immunotherapy is well tolerated and does not lead to excess toxicity [18].

The experience with preoperative RCHT in the setting of trimodal therapy shows reasonable anti-tumor activity [17]. Therefore, we assume that our planned treatment will at least contribute to inhibition of tumor progression. And due to the poor prognosis of this patient population, we would accept the risk of a delay compared to immediate cystectomy or neoadjuvant chemotherapy, given the expected benefit.

In concern of organ specific toxicities, especially gastrointestinal toxicity is a potential risk for patients, which cannot be quantified due to the lack of high quality data in the setting of combined radioimmunotherapy.

Follow-up is limited to 1 year after cystectomy, but we have to emphasis that in this trial the DFS and OS are secondary objectives. Due to the fact, that most recurrence occur in the first year after surgery, the one-year DFS and OS rates as secondary endpoints will provide sufficient data of treatment efficacy in order to plan a consecutive phase III trial.

We will perform this trial to evaluate the combined approach of radiation therapy with a PD-1 checkpoint-inhibitor followed by radical cystectomy to analyze the feasibility of this therapy concept and to generate first efficacy data for a possible future phase III trial.

## Trial status

The trial has started recruitment in February 2019.

## Data Availability

Not applicable.

## Abbreviations

CR: Complete response
CSS: cancer specific survival
CTLA-4: Cytotoxic T-lymphocyte associated protein 4
DFS: Disease free survival
ECOG: Eastern Cooperative Oncology Group
FAS: Full Analysis Set
GCP: Good Clinical Practice
Gy: Gray
ICH: International Conference on Harmonization
MIBC: Muscle-invasive bladder cancer
OV: Overall survival
PD-1: Programmed cell death protein 1
PD-L1: Programmed death ligand-1
PEI: Paul-Ehrlich Institut
RC: Radical cystectomy
RCHT: radiochemotherapy
SOP: Standard Operating Procedure
TMT: Trimodal therapy
